# Enzymatic quantification of total serum bile acids as a monitoring strategy for women with intrahepatic cholestasis of pregnancy receiving ursodeoxycholic acid treatment: a cohort study

**DOI:** 10.1111/1471-0528.15926

**Published:** 2019-09-26

**Authors:** LB Manna, C Ovadia, A Lövgren‐Sandblom, J Chambers, S Begum, P Seed, I Walker, LC Chappell, H‐U Marschall, C Williamson

**Affiliations:** ^1^ Division of Women and Children's Health King's College London London UK; ^2^ Department of Laboratory Medicine Karolinska Institute Stockholm Sweden; ^3^ Women's Health Research Centre Imperial College Healthcare NHS Trust London UK; ^4^ Clinical Biochemistry Frimley Health NHS trust Wexham Park Hospital Slough UK; ^5^ Department of Molecular and Clinical Medicine Sahlgrenska Academy University of Gothenburg Gothenburg Sweden

**Keywords:** Bile acid assay, cholestasis, pregnancy, ursodeoxycholic acid

## Abstract

**Objective:**

To evaluate enzymatic total serum bile acid quantification as a monitoring strategy for women with intrahepatic cholestasis of pregnancy (ICP) treated with ursodeoxycholic acid (UDCA).

**Design:**

Cohort.

**Setting:**

One UK university hospital.

**Population:**

29 ICP cases treated with UDCA.

**Methods:**

Serial samples were collected prospectively throughout gestation. Total serum bile acids were measured enzymatically and individual bile acids by high‐performance liquid chromatography‐tandem mass spectrometry. Data were log‐transformed and analysed with random effects generalised least square regression.

**Main outcome measures:**

The relationship between enzymatic total bile acid measurements and individual bile acid concentrations after UDCA treatment.

**Results:**

In untreated women, cholic acid was the principal bile acid (51%) and UDCA concentrations were <0.5%, whereas UDCA constituted 60% (IQR 43–69) of serum bile acids following treatment and cholic acid fell to <20%. Changes in the total bile acid measurement reflected similar alterations in the concentrations of the pathologically elevated bile acids, e.g. a two‐fold increase in enzymatic total bile acids is accompanied by approximately a two‐fold increase in cholic acid and chenodeoxycholic acid at most UDCA doses (*P* < 0.001). Most of the effects of UDCA on cholic acid occur in the first week of treatment (60% relative reduction, *P* = 0.025, 95% CI 0.2–0.9, from 10 micromol/l (4.7–17.6) to 3.5 micromol/l (1.4–7.5).

**Conclusion:**

Ursodeoxycholic acid becomes the main component of the bile acid measurement after treatment. Enzymatic total bile acid assays are good predictors of both cholic acid and chenodeoxycholic acid, the primary bile acids that are raised prior to treatment.

**Tweetable abstract:**

Ursodeoxycholic acid constitutes approximately 60% of the bile acid measurement and reduces pathological cholic acid in treated women.

## Tweetable abstract

Ursodeoxycholic acid constitutes approximately 60% of the bile acid measurement and reduces pathological cholic acid in treated women.

## Introduction

Intrahepatic cholestasis of pregnancy (ICP) is a liver disease characterised by pruritus and abnormal liver function^1^, ^2^ that affects approximately 0.7% of pregnancies. Increased serum bile acid (BA) concentrations are diagnostic,^3^ with cholic acid (CA) and chenodeoxycholic acid (CDCA) being the main BA species to rise in maternal serum. Liver enzymes are also often elevated.^1^, ^2^

Intrahepatic cholestasis of pregnancy is a relatively benign condition for the mother, as it typically resolves rapidly after delivery.^1^ However, ICP is associated with adverse pregnancy outcomes including spontaneous preterm birth, meconium‐stained amniotic fluid, neonatal unit admission, and stillbirth.^4^, ^5^

The aetiology of ICP is complex, with genetic, environmental, and hormonal factors.^6^ Poor fetal outcomes are thought to occur due to the accumulation of maternal BA in the fetal compartment.^4^, ^7^, ^8^ A large, prospective cohort study showed that fetal complications occurred when maternal serum BA levels (measured enzymatically) were ≥40 micromol/l, increasing by 1–2% for each additional micromol/l elevation.^5^ A recent meta‐analysis confirmed that the risk of spontaneous preterm birth increases when maternal serum BA concentrations are ≥40 micromol/l, and the risk of stillbirth rises with concentrations ≥100 micromol/l.^9^ With the prognostic importance of serum BA concentrations becoming established, UK guidelines recommend that they should be monitored weekly after ICP is diagnosed.^10^

Several techniques exist for serum BA quantification.^11^ Commercially available enzymatic assays based on 3‐α‐hydroxysteroid dehydrogenase are commonly used due to their convenience.^11^, ^12^ However, there is debate about the utility of enzymatic assays as they also measure UDCA in women receiving treatment, possibly due to *in vivo* conversion to *iso‐*UDCA. UDCA is recommended as the first‐line treatment for ICP in European guidelines,^10^, ^13^ and is commonly used by UK obstetricians.^14^ Its use is associated with improvement of maternal symptoms,^15^, ^16^, ^17^, ^18^ as well as reduction of BA, transaminase,^5^, ^16^, ^17^ and CA concentrations^16^ in some studies. However, the recent PITCHES trial that compared the impact of UDCA and placebo on a composite outcome in ICP did not report a reduction in BAs.^35^

This study aimed to assess whether enzymatic assays for total BA quantification can be used for ICP monitoring during UDCA treatment. Using serial samples from opportunistically recruited women at a UK university hospital, we first analysed how serum BAs respond to UDCA treatment by investigating the proportion of individual BAs in the total BA measurement before and after UDCA administration. We then assessed whether total BA concentrations measured by an enzymatic method correlate with changes in CA and CDCA concentrations, the principal BAs to rise in ICP. Finally, we investigated whether any adjustments to the total BA measurements could be applied to account for UDCA enrichment and accurately reflect changes in CA and CDCA.

## Materials and methods

### Participants

Serial blood samples were prospectively collected at timed intervals throughout pregnancy from 51 women diagnosed with ICP and opportunistically recruited from a UK hospital (Queen Charlotte's and Chelsea Hospital). Only women who were recruited prior to commencing UDCA treatment, and who therefore provided serum samples both before and after UDCA treatment, were included in the study (*n* = 29). ICP was diagnosed in women with pruritus with no other identifiable cause, and serum BAs of 14 micromol/l or greater. The majority of women also had elevated alanine aminotransferase (ALT), but this was not required for diagnosis. UDCA treatment was commenced after diagnosis according to the preference of the woman and her practitioner. Between two and eight samples were taken from each woman at varying intervals. A summary of the study design can be found in Figure S1. Women were excluded from the study if they had other causes of hepatic dysfunction such as haemolysis, elevated liver enzymes and low platelets (HELLP) syndrome; preeclampsia; acute fatty liver of pregnancy; acute viral hepatitis; primary biliary cirrhosis; multiple pregnancy or any cause of biliary obstruction on ultrasound. Patients were not directly involved in the development of the study, but the patient charity (ICP Support) is supportive of the work and the Chief Executive Officer of the charity is a co‐author of the study and helped with patient recruitment and acquisition of data. Pregnancy and fetal outcomes were not investigated in this study, therefore no core outcome sets were used.

### Biochemical analysis

All total serum BA were measured using a commercially available enzymatic assay at Imperial College Healthcare NHS Trust (Total Bile Acids Assay Kit, Diazyme, Diazyme Laboratories, Poway, CA, USA). Concentrations of individual BA species were measured by high‐performance liquid chromatography‐tandem mass spectrometry (HPLC‐MS/MS) as previously described.^19^ The proportions of CA, chenodeoxycholic acid (CDCA), deoxycholic acid (DCA), lithocholic acid (LCA), and UDCA were calculated with reference to the sum of all individual BA concentrations.

### Statistics

Log transformations were used in all datasets due to non‐normally distributed data and results are presented as ratios of the geometric mean values. Log base 2 was used for total bile acids. Results were adjusted for repeated measures (clustering by patient) using random‐effects generalised least squares (RE‐GLS) regression. Trend tests were performed with RE‐GLS with all standard errors adjusted for clustering by patient (repeated measures). Statistical significance was taken as *P* ≤ 0.05. All data used for this analysis are included in the manuscript.

## Results

### Proportion of main bile acid species in treated and untreated women with ICP

To establish the contribution of UDCA to total BA concentrations in maternal serum after UDCA treatment, the proportion of each individual BA was compared in serum samples collected before and after UDCA commencement. The sums of conjugated and unconjugated forms of each BA species were used (Table 1).

**Table 1:** The proportion of individual bile acids measured by HPLC‐MS/MS in women with ICP

BA (%)	Not on UDCA	On UDCA	Fold change in proportion	*P*‐value	95% CI
CA	51.4 (36.1–63.0)	18.7 (12.6–26.2)	0.42	<0.001	0.34–0.52
CDCA	24.9 (20.2–34.7)	13.2 (9.8–19.1)	0.55	<0.001	0.46–0.65
DCA	17.5 (4.0–26.4)	4.9 (1.9–10.1)	0.45	<0.001	0.34–0.60
LCA	0.5 (0.2–1.6)	0.8 (0.4–1.6)	1.40	0.059	0.99–1.99
UDCA	0.3 (0.0–0.9)	60.0 (42.8–69.0)	96.70	<0.001	64.18–145.72

BA, bile acid; CA, cholic acid; CDCA, chenodeoxycholic acid; CI, confidence interval; DCA, deoxycholic acid; LCA, lithocholic acid; UDCA, ursodeoxycholic acid.

Results shown as median (IQR). Values represent percentages of the total bile acid pool. Concentrations of individual BA species were measured by high‐performance liquid chromatography‐tandem mass spectrometry (HPLC‐MS/MS) and proportions calculated with reference to the sum of all individual BA concentrations. Both conjugated and unconjugated bile acid species were used for calculations.

The proportion of CA in serum decreased to 42% of the pretreatment value (95% CI 34–52%, *P* < 0.001) following UDCA treatment, with the median falling from 51% to 19%. CDCA similarly decreased by 55% (*P* < 0.001, 95% CI 46–65%), with the median falling from 25% to 13%. The proportion of DCA also decreased by 45% with UDCA treatment (*P* < 0.001, 95% CI 34–60%), with the median reducing from 18% to 5%. In contrast, the proportion of UDCA increased by 97‐fold (*P* < 0.001, 95% CI 64–146%), with the median increasing from 0.3% to 60%. The proportion of LCA was not significantly changed and remained at very low levels.

### Relation between enzymatic total bile acids, CA, and CDCA during UDCA treatment

We investigated whether enzymatic total BA measurements in UDCA‐treated women can reflect changes in CA and CDCA, the principal BAs to rise in ICP. A two‐fold increase in total BAs is associated with a 2.3 times increase in CA (*P* < 0.001, 95% CI 2.0–2.6) and a 1.8 times increase in CDCA (*P* < 0.001, 95% CI 1.7–2.0). This change is consistent across most UDCA doses, except in the range between 1.25 and 1.5 g of UDCA, when this relation is non‐significant for both CA (change in CA of 1.2, *P* = 0.528, 95% CI 0.6–2.8) and CDCA (change in CDCA of 1.2, *P* = 0.604, 95% CI 0.6–2.6) (Table S1).

### Enzymatic total BA measurements can be adjusted during UDCA treatment to reflect changes in CA and CDCA

We investigated whether any adjustments could be applied to enzymatic total BA measurements during UDCA treatment, so that values could reflect CA and CDCA concentrations in serum despite UDCA enrichment. We first applied random‐effects generalised least squares regression to determine the relation between CA and log values of total BA. This was followed by a calculation to predict how much this estimate should be adjusted in samples treated with UDCA. The same model was applied to CDCA.

We found that 0.805 should be subtracted from the log of total BA when women are receiving UDCA (Table 2) in order to reflect CA concentrations more accurately. Arithmetic calculations showed that this would be equivalent to multiplying total BA results by 0.45 (*P* < 0.001, 95% CI 0.3–0.6). For CDCA, subtracting 0.6 from the log of TBA should be applied, which is equivalent to multiplying results by 0.57 (*P* < 0.001, 95% CI 0.5–0.7).

**Table 2:** Adjustment to total bile acid concentrations to reflect CA and CDCA concentrations in maternal serum in UDCA‐treated women with ICP

	Adjustment to log of total BA			Adjustment to total BA (micromol/l)		
	Subtraction	*P*‐value	95% CI	Ratio	*P*‐value	95% CI
CA	−0.8	<0.001	−1.1 to −0.5	0.45	<0.001	0.3 to 0.6
CDCA	−0.6	<0.001	−0.8 to −0.3	0.57	<0.001	0.5 to 0.7

BA, bile acids; CA, cholic acid; CDCA, chenodeoxycholic acid; CI, confidence interval.

Adjustments to the log of total bile acids and their corresponding adjustments to total bile acid results are shown.

### Temporal analysis of total and individual bile acids during UDCA treatment

**Figure 1:**
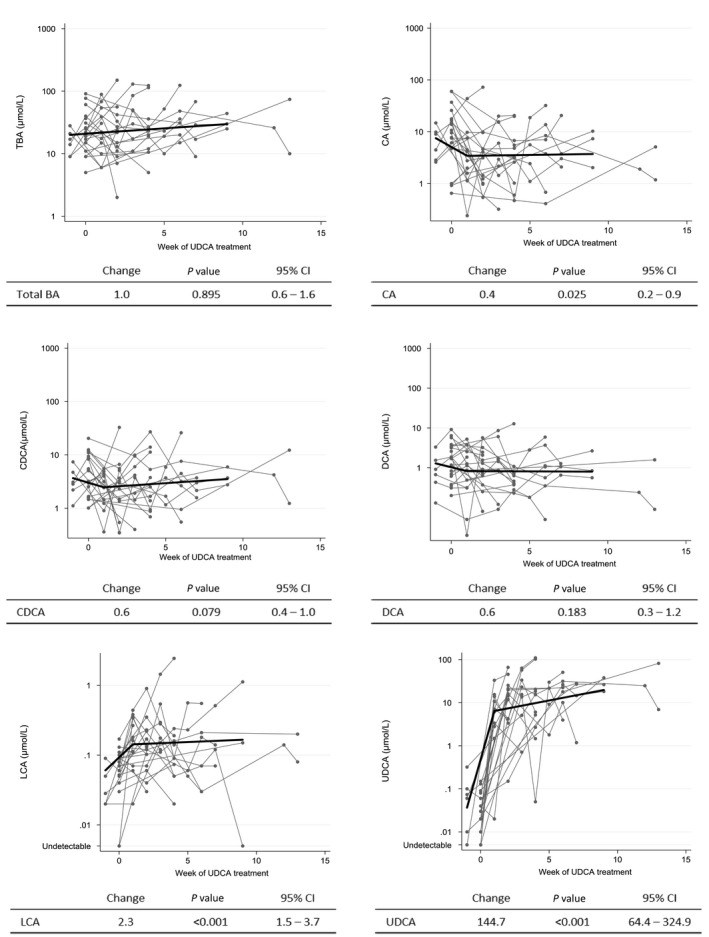
Temporal analysis of total and individual bile acids per week of UDCA treatment. All data were log‐transformed. Week zero of treatment corresponds to the last sample before treatment was commenced. Tables show predicted changes in concentrations, corresponding *P*‐values and 95% confidence interval (CI). CA, cholic acid; CDCA, chenodeoxycholic acid; DCA, deoxycholic acid; LCA, lithocholic acid; TBA, total bile acids; UDCA, ursodeoxycholic acid.

A temporal analysis of BA concentrations was performed in 23 women who had data relating to the start of UDCA. Figure 1 shows the concentrations of total BA and individual BA for each week after commencement of treatment. No evidence of change over time could be established for total BA (change 1.0, *P* = 0.895, 95% CI 0.6–1.6), CDCA (change 0.6, *P* = 0.079, 95% CI 0.4–1.0) or DCA (change 0.6, *P* = 0.183, 95% CI 0.3–1.2). CA was reduced by 65% in the first week of treatment (*P* = 0.025, 95% CI 0.2–0.9), whereas UDCA increased 144 times (*P* < 0.001, 95% CI 64.4–324.9). LCA concentrations also increased (change 2.3, *P* < 0.001, 95% CI 1.5–3.7) but remained at very low levels (Table 3).

**Table 3:** Concentrations of individual bile acids in maternal serum before UDCA treatment and each week after commencing treatment

Weeks	0	1	2	3	4	5	6	7	9	12	13
TBA	21.0 (15–33.8)	22.1 (12.4–46.5)	21.8 (11–33)	39.5 (14.1–67.5)	23.1 (12–36)	21.0 (14.5–37.5)	31.0 (20–48)	23.0 (13–48.5)	30.0 (25–44)	26.0	42.0 (10–74.0)
CA	10.1 (4.7–17.6)	3.5 (1.4–7.5)	4.1 (1.2–4.7)	4.5 (1.8–15.4)	3.3 (1.0–6.8)	4.0 (1.8–12.2)	7.0 (0.7–13.7)	3.4 (2.6–12.2)	7.3 (2.0–10.2)	1.9	3.1 (1.2–5.1)
CDCA	4.1 (1.6–9.1)	3.6 (1.3–4.6)	3.0 (1.4–5.1)	3.9 (1.7–7.7)	2.5 (0.9–11.3)	2.7 (1.4–4.8)	3.2 (0.9–7.6)	2.6 (1.9–3.4)	3.7 (2.8–5.9)	4.2	6.7 (1.2–12.2)
DCA	1.7 (0.7–3.4)	1.0 (0.3–2.2)	0.9 (0.6–2.1)	1.5 (0.5–3.9)	0.5 (0.4–0.7)	1.5 (0.2–2.8)	1.0 (0.4–3.7)	1.1 (0.9–1.2)	0.8 (0.6–2.7)	0.2	0.8 (0.1–1.6)
LCA	0.1 (0.0–0.1)	0.2 (0.1–0.3)	0.1 (0.1–0.2)	0.2 (0.1–0.4)	0.1 (0.0–0.2)	0.1 (0.1–0.4)	0.1 (0.0–0.2)	0.1 (0.1–0.3)	0.1 (0.0–1.1)	0.1	0.1 (0.1–0.2)
UDCA	0.0 (0.0–0.1)	6.5 (2.1–12.3)	14.5 (4.2–21.2)	19.2 (9.3–45.3)	15.3 (2.7–20.5)	15.4 (5.5–25.8)	22.4 (10.1–31.4)	20.7 (7.9–27.5)	26.3 (18.2–37.9)	24.8	44.4 (6.9–1.8)

CA, cholic acid; CDCA, chenodeoxycholic acid; DCA, deoxycholic acid; LCA, lithocholic acid; TBA, total bile acids; UDCA, ursodeoxycholic acid.

Week zero corresponds to the last sample taken before UDCA was commenced. Results shown as median (IQR) and concentrations in micromol/l.

## Discussion

### Main findings

We have demonstrated that UDCA is the predominant BA in the serum of women with ICP receiving UDCA treatment, representing approximately 60% of the total BA measurement. UDCA enrichment is accompanied by a significant decrease in CA and CDCA proportions. We also demonstrate that enzymatic quantification of total BAs is a good predictor of both CA and CDCA concentrations, as a two‐fold increase in total BAs corresponds to approximately a two‐fold increase in both species. Therefore, if a clinician wants to estimate combined CA and CDCA concentrations in a total bile acid measurement while accounting for UDCA enrichment, it is reasonable to reduce the total BA concentrations by 50–60%. A temporal analysis showed that although no predictions can be made for total BA concentrations during UDCA treatment, CA is expected to decrease significantly by approximately 65% in the first week of treatment.

### Strengths and limitations

This study will be of value to clinicians managing women with ICP, as it provides data that allow a better interpretation of enzymatic total BAs during UDCA treatment, which is currently the method most widely used in clinical practice.

A limitation in our study is the lack of data on whether samples were obtained in the fasting or postprandial state. Total BA measured by enzymatic assay can rise 2‐ to 5‐fold, peaking around 90 minutes after a meal. However, given that maternal serum BA measurements are often performed using random samples in antenatal clinics, our results offer a realistic representation of most clinical settings. Another limitation is the lack of a replication resource. It will be important for the data to be repeated in another cohort to confirm the results, in particular to refine the finding that the total BA assay can be reduced by 50–60% to estimate the impact of UDCA treatment on the pathological bile acids, CA and CDCA.

### Interpretation

The establishment of an optimal surveillance strategy for ICP, with the aim of predicting and preventing poor fetal outcomes, is an ongoing challenge.^1^, ^20^, ^21^ The active management of ICP, characterised by increased surveillance towards the end of pregnancy and induction of labour at 37 weeks has become common practice,^14^, ^22^, ^23^, ^24^ particularly for women with severe disease, although the merits of this approach have been debated.^25^, ^26^, ^27^ The RCOG Green Top Guideline^10^ suggests that elective delivery should be discussed with women affected by ICP but does not define this as a management strategy. Nevertheless, a UK survey has shown that 88% of obstetricians induce labour at 37 weeks or earlier, despite the lack of substantial evidence supporting this practice. Some authors propose even earlier delivery, at 36 weeks’ gestation.^24^, ^28^

Bile acids, in particular CA (the principal BA to rise in ICP), has been repeatedly implicated in the pathogenesis of fetal complications. CA has been shown to stimulate myometrial oxytocin receptor expression^29^ and to induce preterm labour when infused into sheep.^30^ Furthermore, addition of CA to the culture medium of rodent and human *in vitro* models of the fetal heart resulted in arrhythmia, suggesting that this BA causes potentially fatal fetal arrhythmia.^31^, ^32^, ^33^ Therefore, the finding of an association between severe ICP (with maternal serum BA ≥40 micromol/l) and fetal complications^4^, ^5^ has influenced clinical practice and increased the focus on monitoring of BA concentrations.^24^, ^34^ If maternal serum BA concentrations are to become established as a decision tool for obstetric interventions, reliable and practical measurement techniques must be used.

There is uncertainty as to whether enzymatic methods of BA measurement can be used in women receiving UDCA treatment. Manufacturers of commercial kits advise against this practice, as this technique quantifies not only endogenous BA but also the ingested UDCA. This leads to the clinical dilemma of whether rises in total BA concentrations after UDCA commencement should be interpreted as a consequence of the drug or as due to a true worsening of ICP.

This study has provided data that will assist interpretation of enzymatic total BA assays in UDCA‐treated women. First, we show that UDCA constitutes approximately 60% of the total BA measurement. Moreover, we show that changes in enzymatic total BA measurements have approximately a 1:1 relation with changes in CA and CDCA, which indicates that this technique is a good predictor of both BAs. Further calculations show that to use enzymatic total BAs as a predictor of CA and CDCA concentrations in serum following commencement of UDCA treatment, total BA concentrations can be reduced by 50–60%.

Furthermore, our temporal analysis provides data on when UDCA effects should be expected. Although no predictions can be made regarding total BAs during treatment, reinforcing the heterogeneous nature of ICP, CA is typically reduced by 65% in the first week after starting UDCA treatment. Therefore, even during the initial period of UDCA enrichment, a sharp or persistent increase in total BAs most likely indicates deteriorating disease.

## Conclusion

Ursodeoxycholic acid constitutes the majority of the BA measurement of women with ICP on UDCA treatment. Total BAs measured enzymatically are good predictors of underlying changes in CA and CDCA. An adjustment of 50–60% can be applied to total BA concentrations to reflect both of these BA species.

### Disclosure of interests

No conflicts of interest, financial or otherwise, are declared by the authors. Completed disclosure of interest forms are available to view online as supporting information.

### Contribution to authorship

CW, CO, and LBM designed study; CO, JC, ALS, and LBM collected data; HUM, ALS, LBM, and CO analysed data; SB and PS provided statistical advice; LBM, CO, PS, and CW interpreted results; LBM drafted the manuscript; CW, CO, IA, LCC, HUM, JC, and IW revised manuscript; CW approved the final version of manuscript.

### Details of ethics approval

Women gave written informed consent and the study was carried out in compliance with the 1975 Declaration of Helsinki Guidelines. Permission was obtained from the ethics Committees of Hammersmith Hospitals NHS Trust (97/5197 and 08/H0707/21). The first approval was given in 1997 and the second in 2008.

### Funding

Supported by the Wellcome Trust (grant P30874); Tommy's Charity, ICP Support, the National Institute of Health Research Biomedical Research Centre at Guy's and St Thomas NHS Foundation Trust and King's College London. The views expressed are those of the author(s) and not necessarily those of the NHS, NIHR or the Department of Health.

CW, CO, and JC are funded by the National Institute of Health Research Biomedical Research Centres at Guy's and St Thomas’ Foundation Trust and King's College London and Imperial College Healthcare NHS Trust, the Wellcome Trust, Tommy's Charity, Genesis Research Trust, ICP Support, and the Guy's and St Thomas’ Charity.

## Supporting information


**Figure S1.** Summary of study design.

**Table S1.** Relation between a 2‐fold increase in total bile acid concentrations on the CA and CDCA concentrations in the serum of women with ICP taking different doses of UDCA.
