# Supplementary material for: Enzymatic quantification of total serum bile acids as a monitoring strategy for women with intrahepatic cholestasis of pregnancy receiving ursodeoxycholic acid treatment: a cohort study
Source: BJOG. 2019 Sep 26;126(13):1633–40. doi: 10.1111/1471-0528.15926 (PMC6899621; doi:10.1111/1471-0528.15926)
Supplement: Supplementary file 1 — Figure S1. Summary of study design. [file BJO-126-1633-s001.pdf]

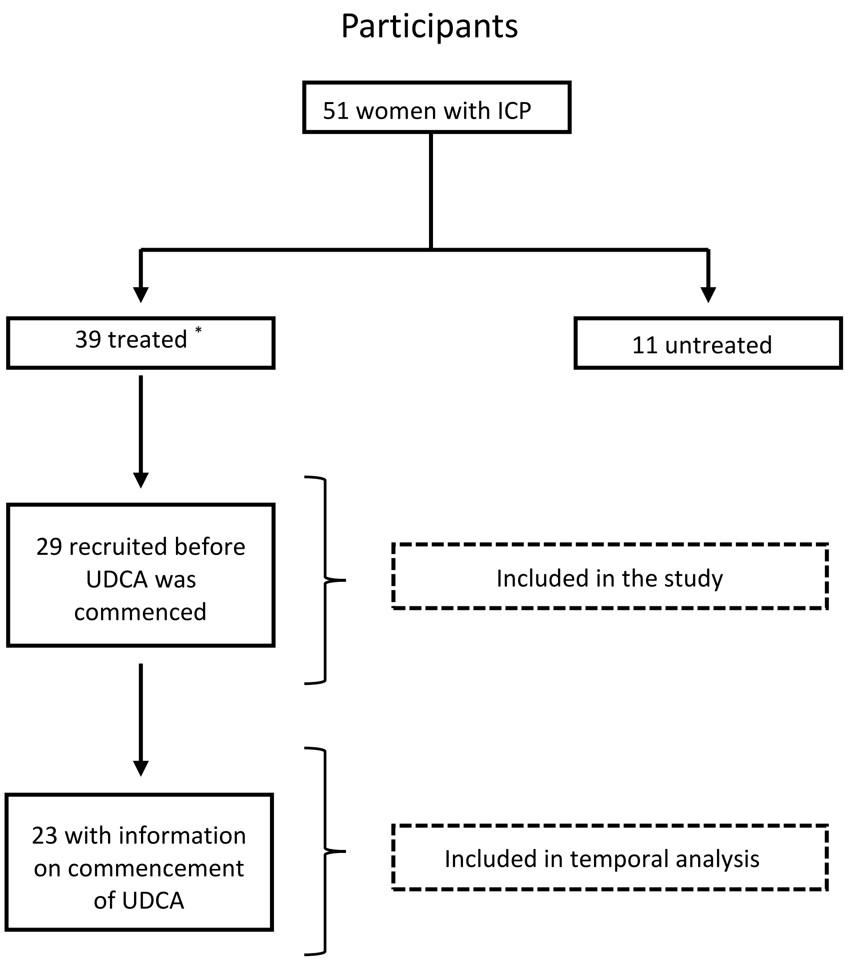

Figure S1. Summary of study design.

51 women with ICP were recruited. 39 women received treatment; 29 of whom were recruited before treatment was commenced. 23 of these women had dates of UDCA commencement documented, and therefore were included in the temporal analysis of total and individual bile acids.
