# Supplementary material for: Enzymatic quantification of total serum bile acids as a monitoring strategy for women with intrahepatic cholestasis of pregnancy receiving ursodeoxycholic acid treatment: a cohort study
Source: BJOG. 2019 Sep 26;126(13):1633–40. doi: 10.1111/1471-0528.15926 (PMC6899621; doi:10.1111/1471-0528.15926)
Supplement: Supplementary file 2 — Table S1. Relation between a 2‐fold increase in total bile acid concentrations on the CA and CDCA concentrations in the serum of women with ICP taking different doses of UDCA. [file BJO-126-1633-s002.pdf]

Table S1. Relationship between a 2-fold increase in total bile acid concentrations on the CA and CDCA concentrations in the serum of women with ICP taking different doses of UDCA. Results shown the effect of doubling the total bile acid concentrations and the change in CA and CDCA. Results obtained with random effects generalised least square regression using log(2) of total bile acids.

	CA			CDCA		
**UDCA dose (g)**	**Change**	**p value**	**95% CI**	**Change**	**p value**	**95% CI**
0	2.3	<0.001	2.0 – 2.6	1.8	<0.001	1.7 – 2.0
0.5 to 1.0	2.1	<0.001	1.8 – 2.5	2.1	<0.001	1.8 – 2.4
1.25 to 1.5	1.3	0.528	0.6 – 2.8	1.2	0.604	0.6 – 2.6
1.75 to 2.0	2.1	<0.001	1.4 – 3.0	2.2	<0.001	1.6 – 2.9

UDCA: ursodeoxycholic acid; CA: cholic acid; CDCA: chenodeoxycholic acid; CI: confidence interval.
